# Social Support Modulates Stress-Related Gene Expression in Various Brain Regions of Piglets

**DOI:** 10.3389/fnbeh.2016.00227

**Published:** 2016-11-29

**Authors:** Ellen Kanitz, Theresa Hameister, Armin Tuchscherer, Margret Tuchscherer, Birger Puppe

**Affiliations:** ^1^Institute of Behavioural Physiology, Leibniz Institute for Farm Animal Biology (FBN)Dummerstorf, Germany; ^2^Institute of Genetics and Biometry, Leibniz Institute for Farm Animal Biology (FBN)Dummerstorf, Germany; ^3^Behavioural Sciences, Faculty of Agricultural and Environmental Sciences, University of RostockRostock, Germany

**Keywords:** social deprivation, social buffering, HPA axis, limbic brain regions, mRNA expression, pig

## Abstract

The presence of an affiliative conspecific may alleviate an individual’s stress response in threatening conditions. However, the mechanisms and neural circuitry underlying the process of social buffering have not yet been elucidated. Using the domestic pig as an animal model, we examined the effect of a 4-h maternal and littermate deprivation on stress hormones and on mRNA expression of the glucocorticoid receptor (GR), mineralocorticoid receptor (MR), 11ß-hydroxysteroid dehydrogenase (11ß-HSD) types 1 and 2 and the immediate early gene c-fos in various brain regions of 7-, 21- and 35-day old piglets. The deprivation occurred either alone or with a familiar or unfamiliar age-matched piglet. Compared to piglets deprived alone, the presence of a conspecific animal significantly reduced free plasma cortisol concentrations and altered the MR/GR balance and 11ß-HSD2 and c-fos mRNA expression in the prefrontal cortex (PFC), amygdala and hypothalamus, but not in the hippocampus. The alterations in brain mRNA expression were particularly found in 21- or 35-day old piglets, which may reflect the species-specific postnatal ontogeny of the investigated brain regions. The buffering effects of social support were most pronounced in the amygdala, indicating its significance both for the assessment of social conspecifics as biologically relevant stimuli and for the processing of emotional states. In conclusion, the present findings provide further evidence for the importance of the cortico-limbic network underlying the abilities of individuals to cope with social stress and strongly emphasize the benefits of social partners in livestock with respect to positive welfare and health.

## Introduction

In social species, one of the most profound moderators of function in the hypothalamic-pituitary-adrenal (HPA) axis during times of stress may be the presence of significant social partners (Hennessy, 1997; DeVries et al., 2003). This model of social support, also known as social buffering, postulates that during times of stress, the presence of significant social partners down-regulates activity in the HPA axis and serves to buffer the individual’s stress response against stressful stimuli (Cohen and Wills, 1985). The positive effects of social support on the physiological and behavioral responses to stress are well demonstrated and have been described in a wide range of species, including humans, non-human primates, rodents and farm animals (Kikusui et al., 2006; Hennessy et al., 2009; Rault, 2012). An understanding of the underlying neurobiological and psychological mechanisms of this buffering is only beginning to emerge, but an important part of this research concerns the neurocircuitry that regulates the activity of the HPA axis.

Because the brain is the key organ in reacting to and coping with stress, as well as in the recovery process, a distributed neural circuitry determines what is threatening and stressful to the individual. In addition to the hypothalamus and brain stem, which are essential for autonomic and neuroendocrine responses to stressors, higher cognitive areas of the brain play a key role in memory, anxiety and decision making (McEwen, 2007). In psychologically challenging circumstances, limbic and cortical regions such as the amygdala, hippocampus and prefrontal cortex (PFC) relay information about threats that can activate or terminate stress responses through modulation of the HPA axis (Hostinar et al., 2014). Glucocorticoids are important mediators of stress and their action in the nervous system is primarily regulated via intracellular glucocorticoid receptors (GRs) and mineralocorticoid receptors (MRs) and both isoforms of the glucocorticoid-metabolizing enzymes 11β-hydroxysteroid dehydrogenase (11β-HSD1 and 11β-HSD2; de Kloet et al., 1998, 2005; Holmes and Seckl, 2006). Furthermore, there is increasing evidence to support the hypothesis that the balance of MRs to GRs is crucial for effective regulation of the stress response and for resilience to psychological disorders (Oitzl et al., 2010).

It is well known that social isolation is a severe psychological stressor that affects a number of behavioral and physiological functions in gregarious species, and the impact of social isolation also depends on the developmental stage of individuals (Hall, 1998; Cacioppo et al., 2011; Hawkley et al., 2012). In animal husbandry, there is a growing interest in the assessment of psychosocial stress, such as the disruption of interactions with mother and/or companions at weaning, to improve our understanding of the biological basis of animal welfare. Moreover, the time of weaning in modern pig production has shifted to younger ages which may lead to severe weaning stress and a state of compromised welfare (Hameister et al., 2010). Yet, information on the impact of psychosocial stress on brain development in postnatal pigs and the underlying molecular mechanisms are limited (Poletto et al., 2006; Sumner et al., 2008; Kanitz et al., 2009). Using the pig as a suitable animal model, previous studies have shown that a 4-h period of social isolation in piglets resulted in increased behavioral arousal, HPA axis activation and stress-related gene expression in the brain (Kanitz et al., 2009), as well as altered immune function (Tuchscherer et al., 2009, 2010). However, recent studies have demonstrated that these social deprivation-induced changes in behavior, HPA activity and immune response can be attenuated by social support from age-matched conspecifics that results in positive effects on the welfare and ability to cope with stress in piglets (Kanitz et al., 2014; Tuchscherer et al., 2014). Furthermore, it has been shown that the degree of familiarity may influence the effectiveness of social support; familiar piglets cause a more pronounced buffering effect on stress hormones and active behavior in test situations (Kanitz et al., 2014).

Based on previous findings from behavioral and physiological research, we supposed that specific limbic areas of the brain participate, in an age-related manner, in the social buffering of the glucocorticoid-regulated stress response. To test this assumption, we examined the effects of a social deprivation procedure (4 h) on stress hormones and the mRNA expression of GR, MR, 11β-HSD1 and 11β-HSD2, as well as on expression of the immediate early gene c-fos in the PFC, amygdala, hippocampus and hypothalamus of 7-, 21- and 35-day old piglets that underwent maternal and littermate deprivation, either alone or with a familiar or unfamiliar age-matched piglet. Non-deprived piglets of the same age served as controls.

## Materials and Methods

Animal care and all procedures involving animal treatment followed the regulations and guidelines of the German Law of Animal Protection were approved by the relevant authorities (Landesamt für Landwirtschaft, Lebensmittelsicherheit und Fischerei, Mecklenburg-Vorpommern, Germany; LALLF M-V/TSD/7221.3-2.1-015/05).

### Animals and Experimental Procedure

The 108 piglets used in this experiment were selected from 27 German Landrace litters within three trials, bred and raised in the experimental pig unit of the Leibniz Institute for Farm Animal Biology (Dummerstorf, Germany). After birth the litter size was standardized to 10 piglets. During the suckling period, sows and their piglets were housed in a loose farrowing pen (6 m^2^), with a water-heated lying area and with unrestricted access to food and water, and at a nearly constant room temperature (28 ± 1°C) with controlled lighting (12/12 h light/dark cycle, lights on at 06:00 h). After final stabilization of the “teat order” at 7 days of age (Puppe and Tuchscherer, 1999) and at 21, or 35 days of age, four piglets from each litter were randomly allocated to four groups as described by Tuchscherer et al. (2014, 2016): (1) maternal and littermate deprivation (piglets were separated alone, DA); (2) maternal and littermate deprivation in the presence of a familiar conspecific (i.e., a piglet from the same litter, DF); (3) maternal and littermate deprivation in the presence of an unfamiliar conspecific (i.e., an age-matched piglet from another litter without any previous contacts to the deprived piglet, DU); and (4) no social deprivation (control group, C). The allocation of male and female piglets within the groups was approximately equivalent (*n* = 9 per treatment and age group). No data were collected on accompanying animals, which were 94% of the same sex as the animals examined. The experimental animals were separated from their mother and littermates in separate test rooms located in the same experimental station for one 4-h period in the morning (07:00 h and 11:00 h). During the social deprivation period, the piglets were placed either alone or together with a familiar or unfamiliar age-matched conspecific into a wooden box (0.68 m × 0.75 m × 0.65 m) with sawdust on the floor and adequate air passage. Within the box, the piglets were separated by a fine wire mesh. The socially deprived piglets were kept under the same light intensity, air and temperature conditions as in the farrowing pen. The control piglets remained undisturbed in the farrowing pen during this time.

One hour before (in order to exclude an influence of the blood sampling on the deprivation experiments) and immediately after the social treatment, blood samples for hormone analyses were taken from each piglet within less than a minute while the animals were in a supine position. The samples were collected by anterior vena cava puncture, transferred to ice-cooled polypropylene tubes containing an EDTA solution, placed on ice, and subsequently centrifuged at 2000 g for 15 min at 4°C for plasma extraction. Plasma was then stored at −20°C until analysis for adrenocorticotropic hormone (ACTH), cortisol and corticosteroid-binding globulin (CBG) concentrations. Immediately after the second blood sampling, the piglets were euthanized with an intravenous injection of T61^®^ (embutramide/mebezonium iodide/tetracaine hydrochloride, Intervet, Unterschleißheim, Germany). The brains were quickly removed (<5 min), and the PFC, amygdala, hippocampus, hypothalamus and the pituitary were dissected out of both brain hemispheres on sterile, ice-cold Petri-dishes. The brain tissues were incubated overnight in RNAlater RNA Stabilization Reagent (Qiagen, Hilden, Germany) at 2–4°C to protect RNA integrity and then transferred to −80°C for storage. A previously published stereotaxic atlas of the pig brain served as a reference (Félix et al., 1999).

### Stress Hormone Analyses

ACTH concentrations in 200 μl plasma samples were measured in duplicate by a highly sensitive and specific two-site ELISA assay (DRG Instruments GmbH, Marburg, Germany) according to the manufacturer’s instructions. The assay has been previously validated with porcine plasma (Kanitz et al., 2004). The lowest level of ACTH that can be detected by this assay is 3.3 pg/ml, and the intra- and inter-assay coefficients of variation (CV) were 2.3% and 4.5%, respectively.

Plasma cortisol concentrations were analyzed in duplicate using a commercially available ^125^I-RIA kit (DSL Inc., Sinsheim, Germany) according to the manufacturer’s guidelines, and the assay was validated for use with porcine plasma (Kanitz et al., 2004). The test sensitivity was 8.1 nmol/l, and the intra- and inter-assay CV were 8.2% and 9.8%, respectively.

Plasma samples were assayed for CBG using a modified binding assay previously described by Kanitz et al. (2002, 2012). Briefly, after removing endogenous steroids from the plasma by dextran-coated charcoal treatment, 25 μl of plasma was incubated with 0.78 nM unlabeled cortisol (Hydrocortisone, Merck, Darmstadt, Germany) and 25 pM ^3^H-cortisol (specific radioactivity 68 Ci/mmol, Amersham Pharmacia Biotech, Freiburg, Germany). For determination of non-specific binding, a 100-fold excess of unlabeled cortisol was added. The separation of bound and free ^3^H-cortisol was performed by precipitation with dextran-coated charcoal at 4°C and subsequent centrifugation at 1000 g for 10 min. The intra- and inter-assay CV were 7.8% and 9.1%.

The free cortisol index (FCI) as a surrogate for free cortisol concentration in plasma was calculated using the following formula: FCI = [cortisol]/[CBG].

### RNA Extraction and Quantification of Transcripts

The total RNA from individual PFC, amygdala, hippocampus, hypothalamus and pituitary samples were extracted using an RNeasy Lipid Tissue Kit (Qiagen, Hilden, Germany) according to the manufacturer’s protocol. The concentration of RNA was determined by the absorbance at 260 nm. The RNA purity and integrity were assessed by calculating the 260/280 nm ratio and by electrophoresis using 2% agarose in a TBE buffer (89 mM Tris, 89 mM boric acid, 1 mM EDTA, pH 8.0) and SYBR gold stain (MoBiTec, Göttingen, Germany) at a final concentration recommended by the manufacturer.

The mRNA expression of the *NR3C1* gene encoding the GR, of the *NR3C2* gene encoding the MR, of the *HSD11B1* gene encoding the enzyme 11β-HSD1, of the *HSD11B2* gene encoding the enzyme 11β-HSD2 and of the *C-FOS* gene was monitored using a reverse transcription (RT) with subsequent real-time polymerase chain reaction (PCR) as described previously (Löhrke et al., 2005; Kanitz et al., 2009, 2012). RT was carried out with 500 ng of total RNA using an iScript cDNA synthesis kit (BIO-RAD, München, Germany) following the guidelines of the manufacturer. The resulting cDNA was amplified by real-time PCR (iCycler, BIO-RAD, München, Germany) using an iQ SYBR Green Supermix (BIO-RAD, München, Germany). One microliter of the RT reaction solution was added to 10 μl of PCR mix primed with gene-specific oligonucleotides (TIB MOLBIOL, Berlin, Germany). Based on the published cDNA and gene sequences (GR: accession no. AY779185; MR: accession no. M36074; 11β-HSD1: accession no. NM_008288; 11β-HSD2: accession no. AF414125; c-fos: accession no. AJ132510), the primers were designed to span a corresponding intron and to anneal between 60°C and 70°C. The following primer sequences were used: GR (forward, 5′-GTT CCA GAG AAC CCC AAG AGT TCA-3′; reverse, 5′-TCA AAG GTG CTT TGG TCT GTG GTA-3′), MR (forward, 5′-GTC TTC TTC AAA AGA GCC GTG GAA-3′; reverse, 5′-CTC CTC GTG GAG GCC TTT TAA CTT-3′), 11β-HSD1 (forward, 5′-GGT CAA CTT CAG CTA CGT GGT-3′; reverse, 5′-AGG ACA CAG AGA GTG ATG GAC ACG-3′), 11β-HSD2 (forward, 5′-TGG TAC CCT TGA GAT GAC CAA-3′; reverse, 5′-CAC TGG TCC ACG TTT TTC ACT-3′) and c-fos (forward, 5′-GGG ACA GTC TCT CCT ACT ACC ACT-3′; reverse, 5′-GGT GAG GGG CTC TGG TCT-3′).

PCR was carried out using a hot start (3 min, 94°C; 30 s, 60°C; 45 s, 70°C), 45 additional cycles (10 s, 94°C; 30 s, 60°C; 45 s, 70°C with 5 s added in each cycle) and with a final cycle of 10 s, 94°C; 30 s, 60°C; 7 min, 70°C, corresponding to denaturation, annealing and elongation, respectively. The specificity of the products was assessed using a melting point analysis that started at 60°C and elevated to 90°C (1°C per 10 s) as well as by agarose gel electrophoresis (2%). The oligonucleotide structure was verified by sequencing in a subset of the experiments. The mRNA abundance was calculated using a known concentration of standard oligonucleotides and the amplification efficiency determined by the iCycler. The values are expressed as pg per μg of total RNA. The ratio of MR/GR mRNA was computed (×10^2^).

### Statistical Analysis

Statistical analyses of the data were done with the SAS software for Windows, version 9.3 (Copyright, SAS Institute Inc., Cary, NC, USA). All data were checked for normality of distribution and homogeneity of variance. The data fulfilled these assumptions and were evaluated by analysis of variance (ANOVA) using the MIXED procedure in SAS/STAT software. The model for the measured parameters contained the fixed block effect replicate (1–3), deprivation treatment (C, DF, DU, DA), age (days 7, 21 and 35), sex (male and female) and deprivation treatment × age interaction. Sow was also included as a random effect. Additionally, least square means (LS means) and their standard errors (SE) were computed for each fixed effect in the model, and all pairwise differences between LS means were tested using the Tukey-Kramer procedure. Significance was defined as *p* ≤ 0.05.

## Results

### Stress Hormones

**Figure 1:**
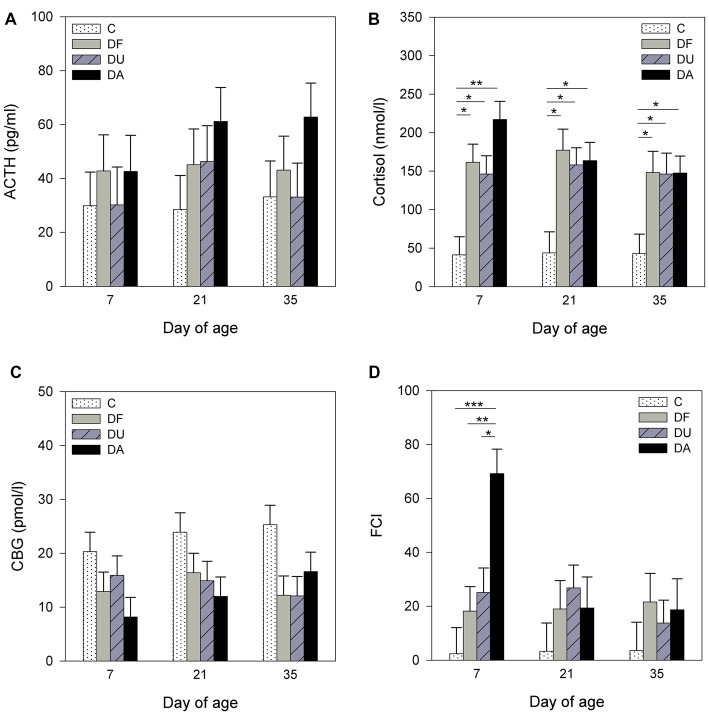
**Effects of conspecific presence on plasma adrenocorticotropic hormone (ACTH; A)**, cortisol **(B)**, corticosteroid-binding globulin (CBG; **C**) concentrations and free cortisol index (FCI; **D**) of piglets during the postnatal period. C: control without social deprivation, DF: social deprivation in the presence of a familiar conspecific, DU: social deprivation in the presence of an unfamiliar conspecific, DA: social deprivation where the piglets were separated alone. Data are expressed as least square means (LS means) ± standard errors (SE). Significant differences are indicated by asterisks (*p < 0.05, **p < 0.01, ***p < 0.001; Tukey-Kramer test).

Before the social deprivation procedure, there were no significant differences observed in plasma ACTH, cortisol or CBG concentrations and FCI among all of the examined piglets (*p* > 0.15; Table 1). ANOVA indicated significant effects of the deprivation treatment on cortisol (*F*_(3,56)_ = 19.90, *p* < 0.001) and CBG (*F*_(3,72)_ = 7.73, *p* < 0.001) concentrations, on the FCI (*F*_(3,69)_ = 5.04, *p* < 0.01) and showed a tendency for an effect on ACTH (*F*_(3,66)_ = 2.58, *p* = 0.06) concentrations. As shown in Figure 1, the results of Tukey-Kramer test revealed significantly higher cortisol concentrations in all socially deprived piglets than in control piglets on day 7 and day 21 (all at least *p* < 0.05), whereas the differences did not reach significance on day 35 (*p* = 0.07). For FCI, statistical significance between the deprivation treatments was only found on day 7. DA piglets displayed a significantly higher FCI than the control (*p* < 0.001), DF (*p* < 0.01) and DU (*p* < 0.05) piglets. There were no significant effects of age (*p* > 0.67), sex (*p* > 0.20) or the deprivation treatment × age interaction (*p* > 0.57) on stress hormones or FCI.

**Table 1:** **Adrenocorticotropic hormone (ACTH), cortisol, corticosteroid-binding globulin (CBG) concentrations and the free cortisol index (FCI) in piglets prior to maternal and littermate deprivation**.

	Social deprivation procedures			
Hormones	C	DF	DU	DA
**ACTH (pg/ml)**
Day 7	12.3 ± 5.1	19.9 ± 5.5	14.1 ± 5.7	15.5 ± 5.4
Day 21	16.9 ± 5.1	27.3 ± 5.4	21.9 ± 5.5	22.4 ± 5.2
Day 35	25.9 ± 5.2	27.1 ± 5.2	27.6 ± 5.2	23.9 ± 5.2
**Cortisol (nmol/l)**
Day 7	37.3 ± 7.9	44.3 ± 7.9	42.2 ± 7.9	45.0 ± 7.9
Day 21	44.9 ± 8.8	57.6 ± 8.8	49.8 ± 9.7	40.9 ± 7.4
Day 35	51.7 ± 9.0	65.8 ± 9.1	41.9 ± 8.4	56.1 ± 7.5
**CBG (pmol/l)**
Day 7	23.5 ± 4.2	20.9 ± 4.2	27.9 ± 4.2	21.8 ± 4.2
Day 21	16.9 ± 4.2	26.7 ± 4.2	17.6 ± 4.2	33.4 ± 4.2
Day 35	25.3 ± 4.3	21.5 ± 4.3	17.9 ± 4.3	23.3 ± 4.3
**FCI**
Day 7	1.8 ± 1.9	2.4 ± 1.8	1.5 ± 1.8	2.1 ± 1.8
Day 21	2.9 ± 2.1	2.6 ± 2.1	3.2 ± 2.3	2.3 ± 1.7
Day 35	2.6 ± 1.9	3.1 ± 2.1	3.3 ± 2.3	2.4 ± 1.7

*C: control without deprivation, DF: social deprivation in presence of a familiar piglet, DU: social deprivation in presence of an unfamiliar piglet, DA: total deprivation/isolation alone. Data are expressed as least square means (LS means) ± standard errors (SE)*.

### Gene Expression in Brain Regions

#### MR/GR Ratio

**Figure 2:**
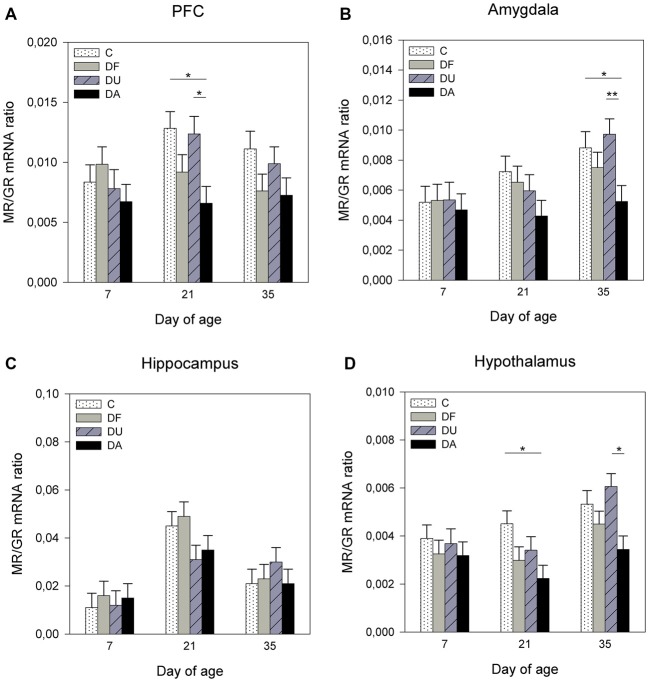
**Effects of conspecific presence on the ratio of mRNA expression of the mineralocorticoid receptor (MR) to the glucocorticoid receptor (GR) in the prefrontal cortex (PFC; A)**, amygdala **(B)**, hippocampus **(C)** and hypothalamus **(D)** of piglets during the postnatal period. C: control without social deprivation, DF: social deprivation in the presence of a familiar conspecific, DU: social deprivation in the presence of an unfamiliar conspecific, DA: social deprivation where the piglets were separated alone. Data are expressed as LS means ± SE. Significant differences are indicated by asterisks (*p < 0.05, **p < 0.01; Tukey-Kramer test).

ANOVA showed treatment effects on the MR/GR mRNA ratio in the PFC (*F*_(3,69)_ = 7.47, *p* < 0.001), amygdala (*F*_(3,71)_ = 4.45, *p* < 0.01) and hypothalamus (*F*_(3,70)_ = 10.03, *p* < 0.001). There was no significant effect of deprivation treatment in the hippocampus (*F*_(3,71)_ = 0.51, *p* = 0.67; Figure 2C). Tukey-Kramer tests indicated a lower MR/GR mRNA ratio in DA piglets than in control and DU piglets in the PFC on day 21 (all *p* < 0.05; Figure 2A), in the amygdala on day 35 (all at least *p* < 0.05; Figure 2B) and in the hypothalamus on day 21 (DA vs. C, *p* < 0.05) and day 35 (DA vs. DU, *p* < 0.05; Figure 2D). Additionally, there was an effect of age on the MR/GR mRNA ratio in the amygdala (*F*_(2,26)_ = 4.09, *p* < 0.05), hippocampus (*F*_(2,26)_ = 9.98, *p* < 0.001) and hypothalamus (*F*_(2,25)_ = 5.04, *p* < 0.05). Tukey-Kramer tests indicated that the MR/GR mRNA ratio was significantly higher in the amygdala on day 35 (0.79 ± 0.06) compared to day 7 (0.53 ± 0.06; *p* < 0.05), in the hippocampus on day 21 (4.00 ± 0.43) compared to day 7 (1.35 ± 0.43; *p* < 0.001) and in the hypothalamus on day 35 (0.48 ± 0.04) compared to day 21 (0.32 ± 0.04; *p* < 0.05). There was no effect of the deprivation treatment × age interaction (*p* > 0.15) or sex (*p* > 0.29) in any brain region investigated.

#### 11β-HSD1

There was no significant influence of the deprivation treatment (*p* > 0.67), sex (*p* > 0.29) or the deprivation treatment × age interaction (*p* > 0.42) on the 11β-HSD1 mRNA expression in the PFC, amygdala, hippocampus or hypothalamus (data not shown). However, ANOVA indicated a significant effect of age on the 11β-HSD1 mRNA expression in the hypothalamus (*F*_(2,29)_ = 4.01, *p* < 0.05) and a tendency in the PFC (*F*_(2,27)_ = 2.66, *p* = 0.09) and amygdala (*F*_(2,24)_ = 3.17, *p* = 0.06). Tukey-Kramer tests revealed higher hypothalamic 11β-HSD1 mRNA expression in the 35-day-old piglets (7.26 ± 0.79 pg/μg total RNA) than in the 21-day-old piglets (4.29 ± 0.75 pg/μg total RNA; *p* < 0.05). A higher 11β-HSD1 mRNA expression was also observed in the amygdala of 35-day-old piglets (1.50 ± 0.15 pg/μg total RNA) than that of 7-day-old piglets (0.97 ± 0.15 pg/μg total RNA; *p* = 0.05).

#### 11β-HSD2

**Figure 3:**
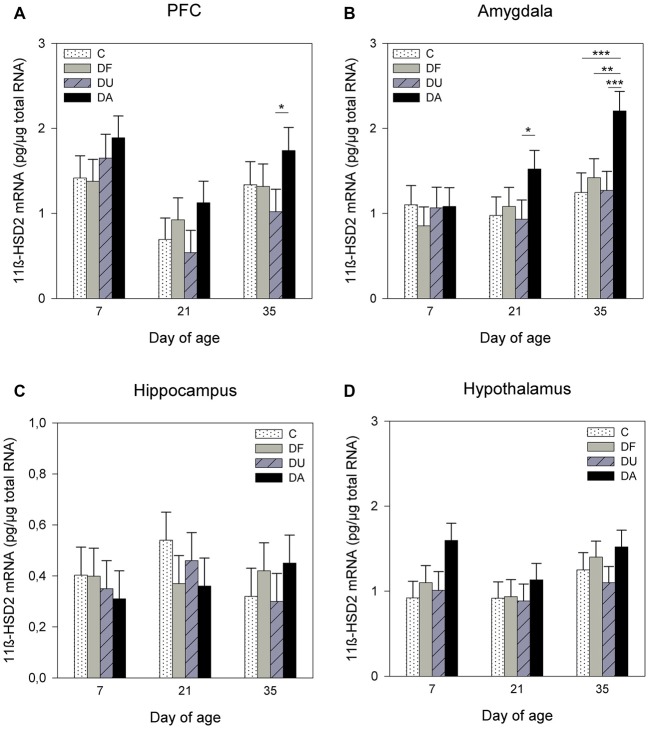
**Effects of conspecific presence on 11β-hydroxysteroid dehydrogenase type 2 (11β-HSD2) mRNA expression in the PFC (A)**, amygdala **(B)**, hippocampus **(C)** and hypothalamus **(D)** of piglets during the postnatal period. C: control without social deprivation, DF: social deprivation in the presence of a familiar conspecific, DU: social deprivation in the presence of an unfamiliar conspecific, DA: social deprivation where the piglets were separated alone. Data are expressed as LS means ± SE. Significant differences are indicated by asterisks (*p < 0.05, **p < 0.01, ***p < 0.001; Tukey-Kramer test).

ANOVA indicated effects of the deprivation treatment on 11β-HSD2 mRNA expression in the PFC (*F*_(3,69)_ = 5.91, *p* < 0.01), amygdala (*F*_(3,71)_ = 7.81, *p* < 0.001) and hypothalamus (*F*_(3,72)_ = 3.57, *p* < 0.05). There was no significant treatment effect in the hippocampus (*F*_(3,71)_ = 0.31, *p* = 0.82). As shown in Figure 3, DA piglets displayed significantly higher 11β-HSD2 mRNA expression than the piglets with social support and the controls in the PFC on day 35 (DA vs. DU, *p* < 0.05; Figure 3A) and amygdala on days 21 (DA vs. DU, *p* < 0.05) and 35 (DA vs. DU, DF, C, all at least *p* < 0.01; Figure 3B). In the hypothalamus, the expression of 11β-HSD2 mRNA tended to have higher values in DA piglets than in control piglets on day 7 (*p* = 0.06; Figure 3D). There was a significant effect of age on the 11β-HSD2 mRNA expression in the PFC (*F*_(2,32)_ = 7.06, *p* < 0.01) and a tendency for 11β-HSD2 mRNA expression to change with age in the amygdala (*F*_(2,29)_ = 2.93, *p* = 0.07), but ANOVA revealed no effects of age in the hippocampus (*F*_(2,35)_ = 0.23, *p* = 0.79) or hypothalamus (*F*_(2,27)_ = 2.41, *p* = 0.11). The Tukey-Kramer test indicated that the mRNA expression of 11β-HSD2 in the PFC was significantly higher in the 7-day-old piglets (1.72 ± 0.21 pg/μg total RNA) than in the 21-day-old piglets (0.77 ± 0.21 pg/μg total RNA; *p* < 0.01). In the amygdala, 7-day-old piglets (0.97 ± 0.15 pg/μg total RNA) tended to have lower 11β-HSD2 expression than 35-day-old piglets (1.50 ± 0.15 pg/μg total RNA; *p* = 0.06). There were no significant effects of the deprivation treatment × age interaction (*p* > 0.28) or sex (*p* > 0.19) in any brain region investigated.

#### c-fos

**Figure 4:**
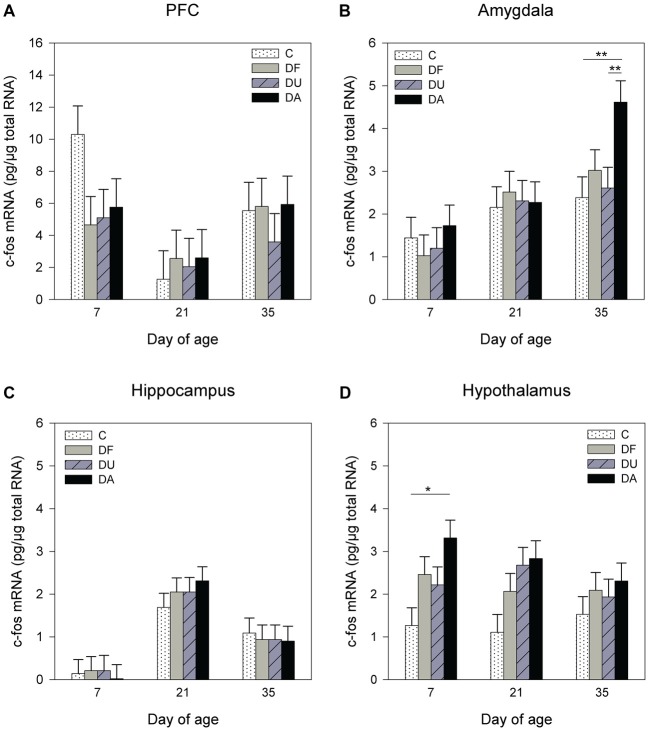
**Effects of conspecific presence on the c-fos mRNA expression in the PFC (A)**, amygdala **(B)**, hippocampus **(C)** and hypothalamus **(D)** of piglets during the postnatal period. C: control without social deprivation, DF: social deprivation in the presence of a familiar conspecific, DU: social deprivation in the presence of an unfamiliar conspecific, DA: social deprivation where the piglets were separated alone. Data are expressed as LS means ± SE. Significant differences are indicated by asterisks (*p < 0.05, **p < 0.01; Tukey-Kramer test).

ANOVA showed significant effects of the deprivation treatment on c-fos mRNA expression in the amygdala (*F*_(3,70)_ = 2.71, *p* = 0.05) and hypothalamus (*F*_(3,71)_ = 8.11, *p* < 0.001) but no effects in the PFC (*F*_(3,93)_ = 0.57, *p* = 0.63; Figure 4A) or hippocampus (*F*_(3,64)_ = 0.25, *p* = 0.86; Figure 4C). A Tukey-Kramer test revealed a higher c-fos mRNA expression in DA piglets than in control and DU piglets in the amygdala on day 35 (all *p* < 0.01; Figure 4B). In the hypothalamus, c-fos mRNA expression exhibit a significant increase and a tendency to increase in DA piglets compared with controls on day 7 (*p* < 0.05), and day 21 (*p* = 0.09), respectively (Figure 4D). In addition, there was an age effect on c-fos mRNA expression in the PFC (*F*_(2,93)_ = 9.72, *p* < 0.001), amygdala (*F*_(2,25)_ = 8.46, *p* < 0.01) and hippocampus (*F*_(2,29)_ = 26.48, *p* < 0.001), but no effect in the hypothalamus (*F*_(2,25)_ = 0.72, *p* = 0.49), and there were no effects of the deprivation treatment × age interaction (*p* > 0.48) in any brain region investigated. Tukey-Kramer tests revealed a higher c-fos mRNA expression in the PFC on day 7 (7.78 ± 0.89 pg/μg total RNA) compared to day 21 (2.25 ± 0.89 pg/μg total RNA; *p* < 0.001), whereas in the amygdala, c-fos mRNA expression was higher on day 35 (3.21 ± 0.33 pg/μg total RNA) compared to day 7 (1.31 ± 0.33 pg/μg total RNA, *P* < 0.001). In the hippocampus, 21- and 35-day-old piglets (day 21: 2.23 ± 0.29 pg/μg total RNA; day 35: 0.96 ± 0.33 pg/μg total RNA) displayed higher c-fos mRNA expression compared to that in 7-day-old piglets (0.15 ± 0.29 pg/μg total RNA, all at least *p* < 0.05). Additionally, there was an effect of sex on the c-fos mRNA expression in the PFC (*F*_(1,93)_ = 5.96, *p* < 0.05). A Tukey-Kramer test indicated that female piglets displayed a higher expression of c-fos mRNA than males (female: 6.36 ± 0.76 pg/μg total RNA; male: 3.79 ± 0.71 pg/μg total RNA; *p* < 0.05).

## Discussion

To our knowledge, this is the first study in postnatal pigs showing that cortico-limbic brain regions are involved in the neural circuits mediating social buffering. The presence of an age-matched conspecific during the period of social deprivation reduced the free cortisol concentration in the plasma and altered corticosteroid receptor and enzyme expression as well as neuronal activity in the PFC, amygdala and hypothalamus.

It is known that social deprivation is a severe stressor with a strong effect on the function of the HPA axis in social species, and on glucocorticoid physiology in social mammals in particular (Hawkley et al., 2012). There is also increasing evidence that social support can decrease the activation of the HPA axis following exposure to a stressor (Hennessy et al., 2000, 2006; Ruis et al., 2001; Bosch et al., 2009). Recently, studies from our group have shown that the presence of an age-matched conspecific can have a direct calming effect on piglets during social deprivation, which was demonstrated by a reduction in stress-induced HPA activity and altered reactions in behavioral situations (Kanitz et al., 2014; Tuchscherer et al., 2014). In the present study, piglets that were socially deprived, either with or without social support, displayed an increase in cortisol and a decrease in CBG concentrations compared to control animals, indicating a greater availability of biologically active cortisol in socially deprived piglets (Le Roux et al., 2002). However, a significant increase in the free cortisol concentration (FCI) was only found in piglets deprived alone on day 7, which is in line with our previous results (Kanitz et al., 2009). It has previously been shown, based on behavioral and some hormonal patterns as well as expression of stress-related genes in brain regions, that younger piglets may have more problems with adapting to stressful life events (Poletto et al., 2006; Hameister et al., 2010). Moreover, replay experiments with the nursing vocalization of sows clearly showed that 1-week-old piglets reacted with a strong approach and contact response indicating their high motivation to gain nutritional and social support. In contrast, the reaction of 5-week-old piglets was rather weak, reflecting their increased independence of direct maternal support (Puppe et al., 2003). Confirming these results, the present study showed that the presence of a conspecific reduced the free cortisol fraction in 7-day-old piglets, whereas no significant effects of deprivation and buffering were found for this parameter in 21- and 35-day-old piglets.

The major pathway in which social stress induces physiological and behavioral effects is the activation of the HPA axis. Circulating glucocorticoids bind to receptors distributed throughout the brain and exert negative feedback inhibition at multiple levels of the HPA axis (Cone et al., 2003). The current evidence also suggests that negative feedback involves corticosteroid receptors in other areas outside the HPA axis, including the PFC and limbic structures such as the hippocampus and amygdala (Oitzl et al., 2010). The present study shows that the social deprivation procedure (4 h) altered the ratio of MR/GR mRNA expression, the glucocorticoid-regulating enzyme 11β-HSD2 and the immediate early gene c-fos in different brain regions and that the presence of an age-matched conspecific may have reversed these alterations. Although we could not identify distinct subnuclei of the PFC, amygdala, or hypothalamus using qPCR, we were able to detect significant differences in corticosteroid receptors, enzymes and c-fos mRNA abundance in these stress-related brain regions between piglets deprived with and without social support and control piglets, which may be caused by a widespread regulation or discrete changes in individual subnuclei. In the brain, MRs and GRs operate in balance, and an imbalance in the MR/GR ratio may compromise overall homeostasis and lead to further HPA dysregulation, which can enhance vulnerability to diseases. Therefore, it has been suggested that the balanced activation of MRs and GRs is crucial for mental health (de Kloet et al., 2005). In the present study, the ratio of MR/GR mRNA was significantly lower in the PFC, amygdala and hypothalamus on day 21 and/or 35 in piglets deprived alone compared to controls, indicating a disturbed receptor balance. Social support from unfamiliar conspecifics significantly reversed the imbalanced MR/GR ratios in the PFC on day 21 and in the amygdala and hypothalamus on day 35, whereas the effects of familiar conspecifics were not statistically significant compared to piglets deprived alone. In a previous study, we found that the presence of familiar partners strengthened the efficiency of the buffering effects on active behavior and stress hormones, whereas alterations in the mRNA expression of hypothalamic corticosteroid receptors were not explicitly affected by the degree of familiarity with the social partner (Kanitz et al., 2014). Here, we again did not find any effect of familiarity on the MR/GR ratios in different brain regions. This may be due to analytical variability and/or the low number of animals in the statistical analysis, but could also refer to different sensory systems which may activate neural and/or hormonal signals to moderate the HPA stress response (Hennessy et al., 2009).

Furthermore, we found an increase in 11β-HSD2 mRNA expression in the PFC on day 35 and in the amygdala on days 21 and 35 in piglets isolated alone compared to that in deprived piglets with social support, whereas no significant effects were found for the expression of 11β-HSD1 mRNA. It is known that 11β-HSD1 regenerates active glucocorticoids from their inactive 11-keto derivatives and is widely expressed throughout the adult central nervous system (CNS). Conversely, 11β-HSD2 is a dehydrogenase that inactivates glucocorticoids (Holmes and Seckl, 2006). The major central effects of 11β-HSD2 are observed during development, as the expression of 11β-HSD2 is high in fetal tissues including the neonate brain, but it is also expressed in a few discrete regions of the adult brain in the hypothalamus, amygdala and hippocampus (Wyrwoll et al., 2011). It is thought that 11β-HSD2 protects MRs from activation by glucocorticoids (Gomez-Sanchez and Gomez-Sanchez, 1992), and therefore, we assume that the increased 11β-HSD2 mRNA expression of DA piglets in specific brain regions may be an adaptive response to social deprivation.

Additionally, the presence or absence of a conspecific is also reflected by neural activity in the amygdala, as assessed by the mRNA expression of the immediate early gene c-fos. Consistent with a previous study (Kanitz et al., 2009), the present study found an increase in c-fos expression in deprived piglets on day 35. Indeed, the presence of an age-matched conspecific suppressed the c-fos mRNA expression in the amygdala. This pattern of neural suppression in pigs is similar to studies in rats in which was shown that social buffering suppresses fear-associated activation of the lateral amygdala (Kiyokawa et al., 2012; Fuzzo et al., 2015). The present results suggest that social buffering may be regulated by different pathways. The presence of a conspecific may buffer stress effects by reducing free cortisol levels or, alternatively, may reduce the impact of cortisol on brain regions by altering glucocorticoid-regulating receptors and enzymes. One potential mechanism through which positive social interactions suppress glucocorticoids may involve the neuropeptide oxytocin (Heinrichs et al., 2003). We assume that the repeated rewards caused by the high frequency of piglets’ suckling bouts during the early postnatal period (Puppe and Tuchscherer, 2000) are able to stimulate the oxytocin release in the offspring, which in turn may affect directly the HPA axis via ACTH suppression (Gibbs, 1986). For example, it was recently shown in 1-month-old beef calves that maternal contact and suckling increased the oxytocin level and decreased the cortisol concentrations compared to bucket-suckling calves (Chen et al., 2015). However, it should be noted that in addition to oxytocin, other biological mediators such as dopamine, serotonin and opioids also participate in the psychophysiological regulation of social buffering, and that many of the brain structures, which are involved in the attenuation of the stress response are not yet developed in early life (Hostinar et al., 2014).

In the present study, we also found age-dependent effects on gene expression in the brain. Younger piglets exhibit lower MR/GR ratios, and 11β-HSD2 and c-fos mRNA expression in the amygdala, hippocampus and hypothalamus compared to that in older piglets, whereas in the PFC, younger piglets displayed higher c-fos and 11β-HSD2 mRNA expression. These results may be due to different postnatal ontogeny of these separate regions of the CNS in pigs as previously described (Kanitz et al., 2011). Additionally, we found an effect of sex on c-fos mRNA expression in the PFC, with a higher neuronal activation in female piglets, which may reflect differences in PFC engagement during the treatment (Snyder et al., 2015). Similarly, Bailey and Silver (2014) found that female rodents showed an increased CRH mRNA expression in the paraventricular nucleus of the hypothalamus in response to psychological stress, indicating a higher activation of brain neural circuits in females than in males.

Interestingly, in the current study, the effects of conspecific presence were most pronounced in the amygdala. There is evidence that the amygdala not only is involved in fear conditioning and emotionally aversive situations but also participates in processing of emotional learning, cognitive functions and positive emotional states (Phelps and LeDoux, 2005; Hooker et al., 2006; Boissy et al., 2007; Kalbe and Puppe, 2010). Related to the present study, we suggest that the presence of a social partner during the social deprivation procedure may contribute to a reappraisal of the situation changing the emotional valence of the isolated piglet from negative to more positive. Hence, the present results in pigs support the role of the amygdala in processing emotional states, and they emphasize the amygdala as an important component of the neural circuitry involved in inducing social buffering from a conspecific. Furthermore, it is important to realize that psychological and physical stressors activate the HPA axis differently. Whereas physical stressors primarily activate the HPA axis at the level of the PVN (Herman et al., 2003), psychological stressors, such as deprivation and isolation, appear to have a more complex control of the HPA axis, with clear implication of the amygdala (Roozendaal et al., 1991; Prewitt and Herman, 1997). Moreover, indirect effects of social buffering on the HPA axis have also been documented for the PFC, which has strong connections with the amygdala and can modulate the PVN (Figueiredo et al., 2003). Neuroimaging studies in rhesus monkeys and humans support the important role of the PFC in social buffering (Rilling et al., 2001; Eisenberger et al., 2007). However, the mechanism through which social support can reduce stress responses is part of ongoing research; a major role for oxytocin has been proposed, as has the involvement of opioids (Kikusui et al., 2006; Hennessy et al., 2009). It should also be noted that social buffering is not limited to HPA activity. There are additional neurobiological systems that could be involved in social buffering of stress, although at present, their role is not yet clearly understood (Younger et al., 2010; Hostinar et al., 2014).

Taken together, the present results indicate that the peripheral glucocorticoid concentration does not necessarily reflect processing in higher brain regions underlying social buffering of stress, though the presence of a conspecific reduced the activity of various stress-regulating brain regions. These findings demonstrate that uncovering the neural circuitry involved in psychosocial stress responses and social support in farm animals is helpful to understand the biological processes underlying animal welfare, and hence may contribute to the development of approaches to improve husbandry and management procedures.

In conclusion, the present study demonstrates that the presence of an age-matched conspecific can alter the mRNA expression of glucocorticoid-regulating genes in the brain caused by a 4-h period of maternal and littermate deprivation in domestic piglets. We showed, in domestic pigs, that the amygdala in particular is involved in the neural circuitry mediating social buffering, indicating the significance of this brain region both for the assessment of social conspecifics as biologically relevant stimuli and for the processing of emotional states. Furthermore, effects of conspecific presence were particularly pronounced in 21- and 35-day-old piglets, which may reflect a sensitive stage in the early ontogenetic development of the investigated brain regions in pigs. Finally, the present findings provide further evidence for the importance of the cortico-limbic network underlying the abilities of individuals to cope with social stress and strongly emphasize the benefits of social partners in livestock practices, and might also have implications for emotional disorders in humans and potential intervention strategies.

## Author Contributions

EK and BP contributed to the conception and design of the study. EK, TH, MT and BP performed the experiments and collected and analyzed the data. AT performed the statistical analyses. All authors interpreted the data. EK wrote the manuscript.

## Funding

This study was supported by a grant of the Deutsche Forschungsgemeinschaft (KA 1266/4-1).

## Conflict of Interest Statement

The authors declare that the research was conducted in the absence of any commercial or financial relationships that could be construed as a potential conflict of interest.
